# Isolation and characterisation of CD9-positive pituitary adult stem/progenitor cells in rats

**DOI:** 10.1038/s41598-018-23923-0

**Published:** 2018-04-03

**Authors:** Kotaro Horiguchi, Ken Fujiwara, Saishu Yoshida, Takashi Nakakura, Ken Arae, Takehiro Tsukada, Rumi Hasegawa, Shu Takigami, Shunji Ohsako, Takashi Yashiro, Takako Kato, Yukio Kato

**Affiliations:** 1 Laboratory for Anatomy and Cell Biology, Department of Health Sciences, Kyorin University, 5-4-1 Shimorenjaku, Mitaka, Tokyo, 181-8612 Japan; 2 Institute for Reproduction and Endocrinology, Meiji University, 1-1-1 Higashi-mita, Tama-ku, Kawasaki, Kanagawa 214-8571 Japan; 3 Division of Histology and Cell Biology, Department of Anatomy, Jichi Medical University School of Medicine, 3311-1 Yakushiji, Shimotsuke, Tochigi, 329-0498 Japan; 4 Department of Anatomy, Graduate School of Medicine, Teikyo University, 2-11-1 Kaga, Itabashi, Tokyo, 173-8605 Japan; 5 Laboratory of Immunology, Department of Health Sciences, Kyorin University, 5-4-1 Shimorenjaku, Mitaka, Tokyo, 181-8612 Japan; 6 Department of Biomolecular Science, Faculty of Science, Toho University, 2-2-1 Miyama, Funabashi, Chiba, 274-8510 Japan; 7 Department of Life Science, School of Agriculture, Meiji University, 1-1-1 Higashi-mita, Tama-ku, Kawasaki, Kanagawa 214-8571 Japan

## Abstract

S100β protein and SOX2-double positive (S100β/SOX2-positive) cells have been suggested to be adult pituitary stem/progenitor cells exhibiting plasticity and multipotency. The aim of the present study was to isolate S100β/SOX2-positive cells from the adult anterior lobes of rats using a specific antibody against a novel membrane marker and to study their characteristics *in vitro*. We found that cluster of differentiation (CD) 9 is expressed in the majority of adult rat S100β/SOX2-positive cells, and we succeeded in isolating CD9-positive cells using an anti-CD9 antibody with a pluriBead-cascade cell isolation system. Cultivation of these cells showed their capacity to differentiate into endothelial cells via bone morphogenetic protein signalling. By using the anterior lobes of prolactinoma model rats, the localisation of CD9-positive cells was confirmed in the tumour-induced neovascularisation region. Thus, the present study provides novel insights into adult pituitary stem/progenitor cells involved in the vascularisation of the anterior lobe.

## Introduction

The pituitary gland plays key roles in the maintenance of homeostasis through the production of hormones. The rodent pituitary gland is composed of three subdivisions: the anterior, intermediate, and posterior lobes. The anterior lobe contains five types of hormone-producing cells, together with non-hormonal cells such as S100β protein-positive (S100β-positive) cells and fenestrated sinusoids (i.e., endothelial cells and pericytes). For sustaining the function of the anterior lobe, a supply of mature cells from stem/progenitor cells maintained in the pituitary niche is important. These stem/progenitor cells have been identified over the last decade by several approaches, such as side-population (SP), sphere-forming, and niche-isolation assays. SP cells consisting of a stem cell population can be enriched from dispersed cells based on differences in their efflux capacity for the dye Hoechst 33,342 using flow cytometry^1^. Chen *et al*. isolated pituitary stem/progenitor cells as an SP enriched in cells showing high expression of *Sca1* (stem cell antigen 1) with sphere-forming capacity from adult mouse anterior lobes^2^. Later, they separated the SP cells into two fractions based on levels of *Sca1* expression, the Sca1^high^ SP and the non-Sca1^high^ SP, and demonstrated that the non-Sca1^high^ SP included cells expressing high levels of the pituitary stem/progenitor marker sex-determining region Y-box 2 (SOX2). These cells also exhibited a multipotent capacity to differentiate into all pituitary endocrine cell lineages^3^. Additionally, Fauquier *et al*. demonstrated that *Sox2*-expressing cells in the dispersed adult mouse anterior lobe generate pituispheres and differentiate into all types of endocrine cells^4^. Gene tracing analysis of *Sox2* confirmed that *Sox2*-expressing cells of both the embryonic and adult pituitary are pituitary stem/progenitor cells^5^.

A stem/progenitor niche has been identified in some organs, such as the intestines, brain, skeletal muscles, skin, and testes^6–8^, and is defined as a microenvironment that maintains stem cells through growth factors, cell surface proteins, and extracellular matrices (ECMs). In the adult anterior lobe, the marginal cell layer (MCL) facing Rathke’s cleft and SOX2-positive cell clusters scattered throughout the parenchyma are proposed to act as primary and secondary stem/progenitor cell niches, respectively^9–11^. The anterior lobe undergoes a substantial growth wave after birth, along with an increase in the number of hormone-producing cells and the emergence of S100β-positive cells. Interestingly, although the MCL niche exists from early pituitary development to the adult pituitary, the parenchymal niche, which originates from the MCL niche, appears only after birth and increases in cell number during the early postnatal pituitary growth wave^12,13^. We previously reported that SOX2-positive stem/progenitor cells of the rat adult anterior lobe constitute multiple populations defined by the existence of S100β protein and others^12,14^. In particular, approximately 83% of SOX2-positive cells in the anterior lobes of adult rats are immunopositive for S100β and approximately 85% of S100β-positive cells are immunopositive for SOX2 (S100β/SOX2-positive cells)^12^. Intriguingly, S100β-positive cells have been shown to have the ability to differentiate into hormone-producing cells and muscle cells^14,15^. To isolate and characterise S100β/SOX2-positive cells in the anterior lobe, Yoshida *et al*.^11,16^ succeeded in isolating dense cell clusters originating from the parenchymal niche, termed parenchymal stem/progenitor cell clusters (PS clusters), by taking advantage of its tight structure, which is resistant to protease treatment; their stemness was confirmed by the presence of several stem/progenitor cell markers and their differentiation capacity in an *in vitro* differentiation assay.

S100β-positive cells were first observed in the anterior lobe approximately four decades ago in the form of folliculo-stellate cells^17^. However, today, S100β-positive cells in the pituitary lobe are known to comprise heterogeneous populations playing multiple biological roles. Itakura *et al*.^18^ conveniently generated transgenic rats that express green fluorescent protein (GFP) under the control of the *S100β* promoter (S100β/GFP-TG rats), allowing for the visualisation of S100β-positive cells and enabling their isolation by fluorescence-activated cell sorting (FACS). We previously developed a method of separating a particular cell population from the rat anterior lobe using an antibody against cluster of differentiation (CD). In this recent study, we achieved the enrichment of thyroid-stimulating hormone (TSH)-producing cells by means of anti-CD90 antibody treatment together with the pluriBead-cascade cell isolation system^19^. In this study, we aimed to adapt this powerful separation system to isolate a subpopulation of S100β-positive cells from the adult rat anterior lobe. Because S100β-positive cells comprise a small population in the parenchyma at postnatal day 5 (P5, early postnatal period) and because the ratio of S100β/SOX2-positive cells among S100β-positive cells is lower at P5 than at P60 (sexually mature)^12^, a comparative microarray analysis of S100β-positive cells from S100β/GFP-TG male rats at P5 and P60 was performed to identify particular CD antigens specific for adult S100β-positive cells. Ultimately, CD9 was identified as a novel marker for a subpopulation of S100β/SOX2-positive cells, and an anti-CD9 antibody was used to isolate S100β/SOX2-positive cells with the pluriBead-cascade cell isolation system, resulting in 23-fold enrichment. Furthermore, we found that a subset of the isolated CD9-positive cells has the potential to differentiate into endothelial cells.

## Results

### Microarray analysis using S100β/GFP-TG male rats (P5 and P60)

**Figure 1:**
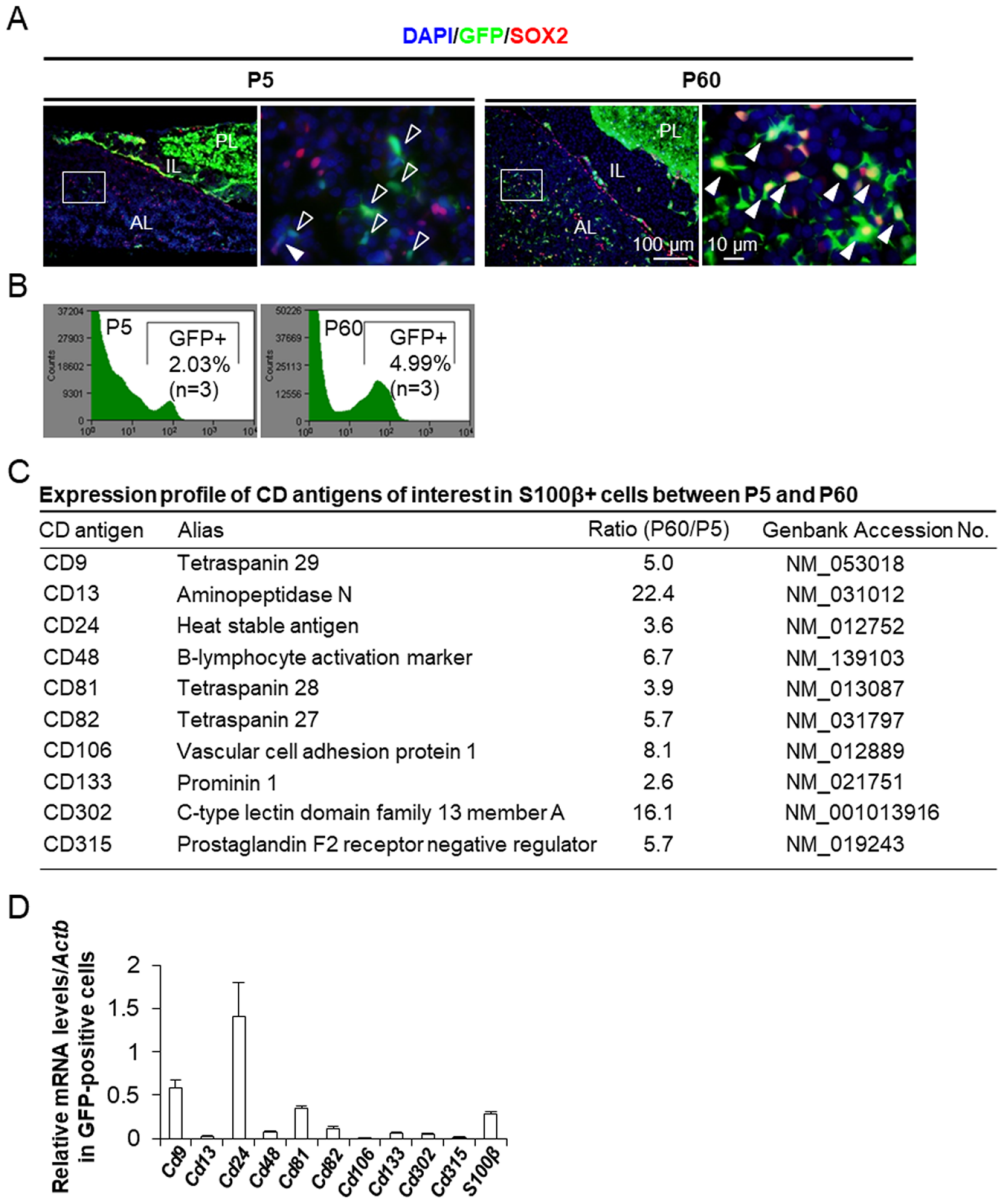
Expression profiles of CD antigens of interest in S100β-positive cells. (**A**) Immunofluorescence staining of SOX2 in the anterior lobe of S100β/GFP-TG rats at P5 and P60. Open white arrowheads indicate S100β-positive cells negative for SOX2. White arrowheads indicate S100β-positive cells positive for SOX2. Right images are high magnifications of boxed areas in left images at P5 and P60. AL, anterior lobe; IL, intermediate lobe; PL, posterior lobe. (**B**) GFP intensity of anterior pituitary cells from S100β/GFP-TG rats at P5 and P60, separated by a cell sorter. Numbers indicate gated cell frequencies (n = 3). (**C**) Comparative expression levels of CD antigens of interest from microarray data of S100β-positive cells at P5 and P60. (**D**) Expression levels of 10 CD genes and *S100β* mRNA in GFP-positive cells from S100β/GFP-TG rats at P60 as determined by qPCR and normalised with an internal control (β-actin gene, *Actb*).

Haematoxylin and eosin (HE) staining and GFP images of frozen sections of the pituitary glands of S100β/GFP-TG male rats at P5 and P60 are shown in Supplementary Fig. S1A. The MCL faces Rathke’s cleft in the anterior and intermediate lobes (Supplementary Fig. S1A). GFP-positive cells were distributed in the anterior, intermediate, and posterior lobes of the pituitary gland. In the posterior lobe, these are pituicytes (Supplementary Fig. S1A). Although GFP-positive cells were also present in the MCL of the intermediate lobe, we focused on the MCL and parenchyma in the anterior lobe in the present study. As shown in Fig. 1A, most S100β/GFP-positive cells in the parenchyma at P5 were immunonegative for SOX2; however, a large number were positive for SOX2 at P60. Dispersed cells from the anterior lobes of S100β/GFP-TG male rats were separated into GFP-positive and -negative cell fractions by a cell sorter (Fig. 1B). We performed a comparative microarray analysis of GFP-positive cells at P5 and P60 to identify CD antigens specific for GFP-positive cells at P60. Ten novel CD antigen genes that were up-regulated (fold change > 2.0) in the GFP-positive fraction at P60 compared with levels at P5 were identified (Fig. 1C). In addition, three CD antigen genes that were down-regulated at P60 (fold change <−2.0) were identified (*Cd90*: NM_012673, *Cd200*: NM_031518, and *Cd231*: NM_00108815). We subsequently performed quantitative polymerase chain reaction (qPCR) to determine the mRNA levels of these 10 genes relative to that of β-actin (*Actb*) in the GFP-positive cell fraction at P60. *Cd9*, *Cd24*, and *Cd81* were clearly expressed in the S100β/GFP-positive cell fraction (Fig. 1D).

### Identification and isolation of CD9-positive cells in the anterior lobe

**Figure 2:**
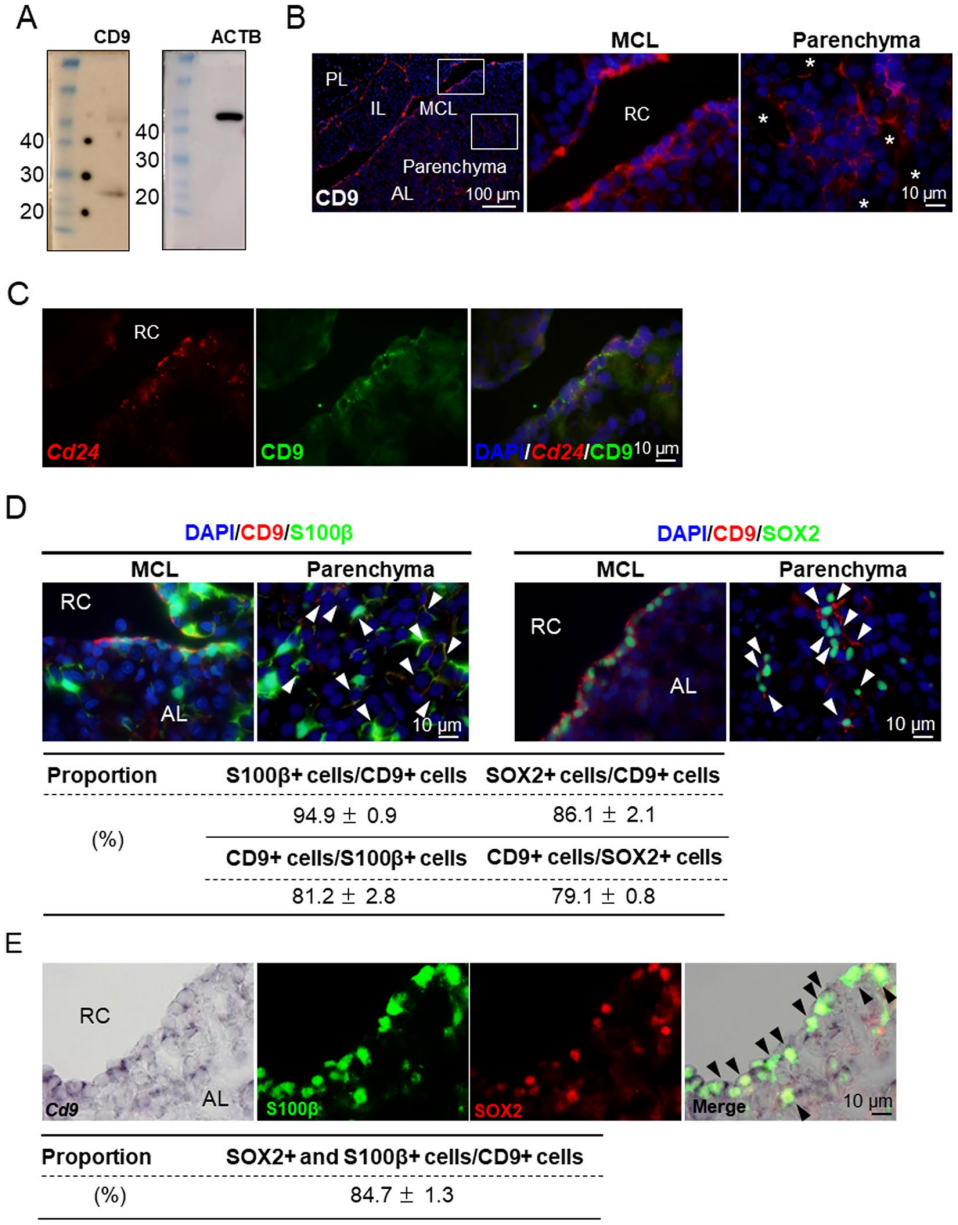
Expression of CD9 in S100β-positive cells. (**A**) Merged images of full-length gels and transfer membranes from western blotting. CD9 (left) and β-actin (ACTB, right) proteins in the anterior lobe as determined from different gels. Molecular markers with their molecular weights (kDa) are indicated in the left lane of each panel. Exposure times were 3 min (CD9) and 2 min (β-actin). (**B**) Immunofluorescence staining of CD9 in the marginal cell layer (MCL) and parenchyma of the anterior lobes of adult male rats. Middle and right images are high magnifications of boxed areas in left images. AL, anterior lobe; IL, intermediate lobe; PL, posterior lobe. RC, Rathke’s cleft. Asterisks indicate blood vessels. (**C**) *In situ* hybridisation of *Cd24* (*red*) and immunohistochemistry of CD9 (*green*). (**D**) Immunofluorescence staining of CD9 (*red*) in the MCL and parenchyma of anterior lobes from adult male S100β/GFP-TG rats and double immunofluorescence for CD9 and SOX2 in the anterior lobes of adult male rats. AL, anterior lobe. RC, Rathke’s cleft. White arrowheads indicate double-positive cells. Lower table shows the proportions of S100β-positive (S100β+) cells among CD9-positive (CD9+) cells, CD9+ cells among S100β+ cells, SOX2-positive (SOX2+) cells among CD9+ cells, and CD9+ cells among SOX2+ cells of the anterior lobe. Numbers of CD9, S100β, and SOX2-positive cells were counted in random areas of the anterior lobe, and the population of each type of cell was calculated for immunohistochemistry. (**E**) *In situ* hybridisation of *Cd9* (left panel) and double immunofluorescence of S100β (*green*) and SOX2 (*red*) in the anterior lobes of adult male rats. Right panels show merged images of the three left panels. AL, anterior lobe. RC, Rathke’s cleft. Black arrowheads indicate triple-positive cells. Lower table shows the proportion of S100β and SOX2-double positive (S100β+ and SOX2+) cells among CD9-positive (CD9+) cells. Numbers of CD9, S100β, and SOX2-triple positive cells were counted in random areas of the anterior lobe, and the ratio of triple-positive cells to CD9+ cells was calculated for *in situ* hybridisation and immunohistochemistry.

We further examined whether anterior lobe cells expressed these candidate 10 genes by *in situ* hybridisation. *Cd9*-expressing cells and *Cd24*-expressing cells were clearly detectable in the MCL of the anterior and intermediate lobes and the parenchyma of the anterior lobe; however, they were undetectable in the posterior lobe (Supplementary Fig. S1B). In contrast, only a few signals were detected for the other eight candidate genes, suggesting that the sensitivity of *in situ* hybridisation was too low to detect these mRNAs. Next, immunohistochemistry was performed using anti-CD9 and anti-CD24 antibodies. The specificity of the anti-rat CD9 antibody was determined by western blot analysis using lysates prepared from the rat adult anterior lobe. An immunoreactive protein band was detected at approximately 25 kDa, which was consistent with the expected size of rat CD9 (Fig. 2A). In contrast, only non-specific immunoreactivity was observed for the anti-CD24 antibody following western blotting and immunohistochemistry (Supplementary Fig. S1C and D). CD9-immunopositive (CD9-positive) cells were localised in the MCL of the anterior and intermediate lobes and the parenchyma of the anterior lobe, but not in the posterior lobe by immunohistochemistry using the anti-CD9 antibody and *in situ* hybridization (Fig. 2B and Supplementary Fig. S1B). Despite the nonspecific immunoreactivity observed in western blotting, immunohistochemistry for CD24 suggested that this protein was localised in the anterior and intermediate lobe. These locations coincided with the results of RNA *in situ* hybridisation, suggesting that this immunoreactivity did not represent nonspecific staining. In the anterior lobe, some CD24-positive cells were immunonegative for S100β (Supplementary Fig. S1D). Based on these results, we selected the anti-CD9 antibody for further study. We performed double-staining using *in situ* hybridisation for *Cd24* and immunohistochemistry for CD9. CD9-positive cells expressed *Cd24* (Fig. 2C), indicating that *Cd24*-expressing cells were the same as CD9-positive cells. CD9 is a member of the tetraspanin superfamily and is a small protein with four transmembrane domains^20,21^. Typically, CD9 forms a complex with CD81, which is also a member of the tetraspanin superfamily and was also detected in the present microarray data (Fig. 1C). Finally, we examined whether CD9-positive cells expressed GFP in S100β-GFP male rats at P5 and P60 by immunohistochemistry. As shown in Fig. S1E, GFP-expressing cells in S100β-GFP male rats were also clearly stained with the anti-CD9 antibody in both the MCL and parenchyma in the anterior lobe at P5 and P60 (Fig. S1E). Most CD9-positive cells expressed GFP and the number of GFP-expressing CD9-positive cells was higher at P60 (Supplementary Fig. S1E). In conclusion, we selected CD9 as a tool to perform the desired function in the present study.

Next, double immunostaining for CD9 with S100β or SOX2 was performed (Fig. 2D). CD9 and S100β-double positive (CD9/S100β-positive) cells and CD9 and SOX2-double positive (CD9/SOX2-positive) cells were present in the rat adult anterior lobe. The proportions of S100β-positive and SOX2-positive cells among CD9-positive cells were 94.9% and 86.1%, respectively (Fig. 2D). In contrast, the proportions of CD9/S100β-positive cells among S100β-positive cells and CD9/SOX2-positive cells among SOX2-positive cells were 81.2% and 79.1%, respectively (Fig. 2D). We finally performed triple-staining using *in situ* hybridisation for *Cd9* and double immunostaining for S100β and SOX2. We observed that triple positive (CD9/S100β/SOX2-positive) cells were present in the rat adult anterior lobe, and the proportion of CD9/S100β/SOX2-positive cells among CD9-positive cells was 84.7% (Fig. 2E).

### Purification of CD9-positive cells localised in the MCL and parenchyma of the adult anterior lobe

**Figure 3:**
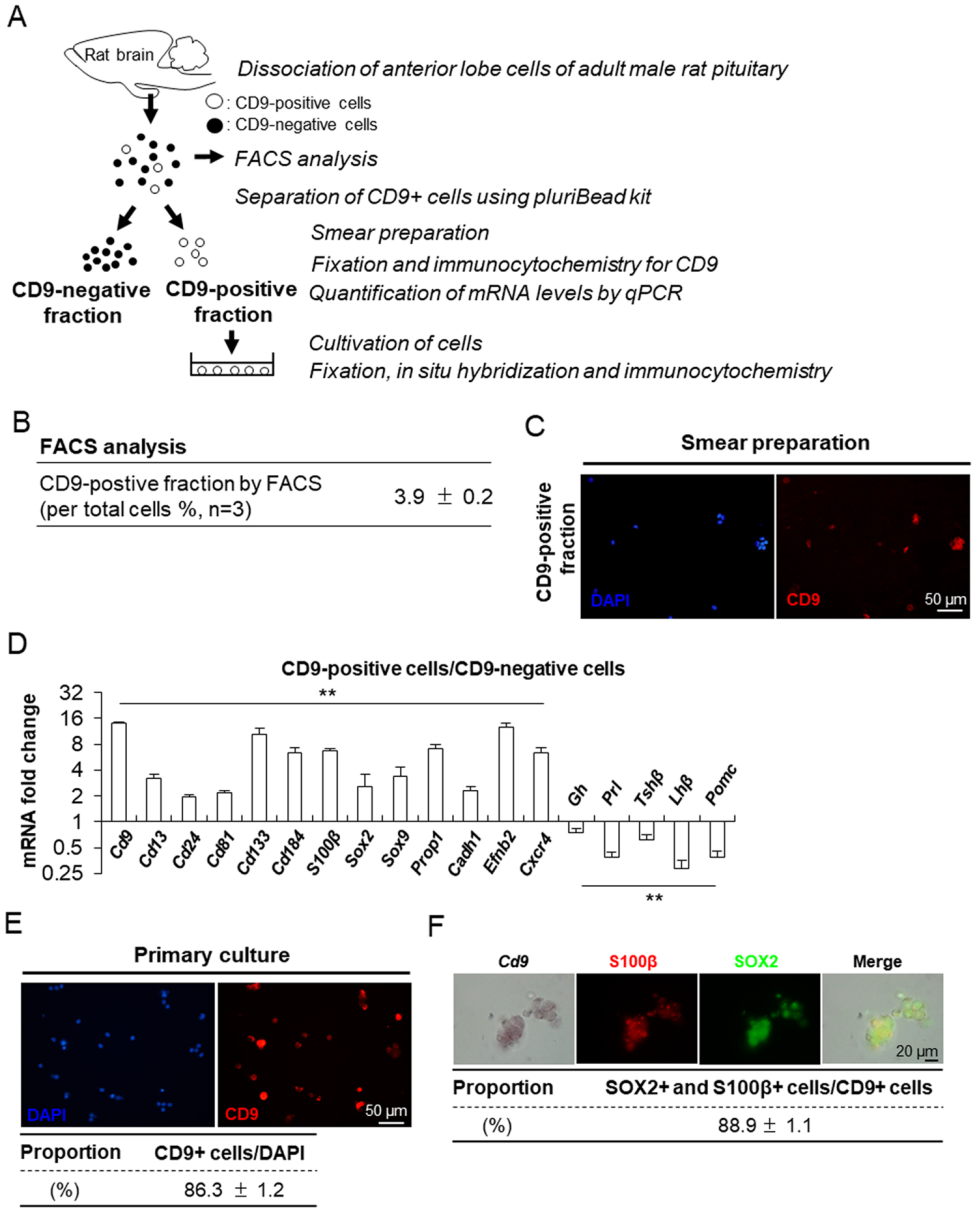
Isolation of CD9-positive cells from the rat anterior lobe. (**A**) Schematic of the experimental steps for FACS analysis, preparation, and cultivation of CD9-positive cells. (**B**) The proportion of CD9-positive cells in rat anterior lobes at P60 estimated by FACS. (**C**) Immunofluorescence staining of CD9 in smear preparations from the CD9-positive fraction. (**D**) mRNA levels of the following genes in CD9-positive and -negative cells were determined by qPCR (mean ± SEM, n = 3), followed by normalisation with an internal control (*Actb*): *Cd9*, *Cd13*, *Cd24*, *Cd81*, *Cd133, S100β*, *Sox2*, *Sox9*, *Prop1*, *Cadh1*, *Efnb2*, *Cxcr4*, *Gh* (growth hormone), *Prl* (prolactin), *Tshβ* (thyroid-stimulating hormone-beta)*, Lhβ* (luteinising hormone-beta), and *Pomc* (proopiomelanocortin). **P < 0.01. The vertical axes are scaled logarithmically. (**E**) Immunofluorescence staining of CD9 in the CD9-positive fraction cultured for 72 h on a non-coated surface with 10% FBS. The proportion of CD9-positive (CD9+) cells in the CD9-positive fraction separated by pluriBeads is indicated (mean ± SEM, n = 3) in the lower row. Numbers of CD9-positive cells were counted in random areas in primary culture wells, and the proportion of each type of cell was calculated. (**F**) *In situ* hybridisation of *Cd9* (left panel) and double immunofluorescence of S100β (*green*) and SOX2 (*red*). The right panel shows a merged image of the three left panels. Numbers of CD9, S100β, and SOX2-triple positive cells were counted in random areas, and the ratio of triple-positive cells to CD9+ cells was calculated for *in situ* hybridisation and immunohistochemistry.

First, we analysed the proportion of CD9-positive cells among rat anterior lobe cells using FACS. We next attempted to purify CD9-positive cells the monoclonal using anti-rat CD9 antibody combined with the pluriBead-cascade cell isolation system (Fig. 3A). The results showed that the proportion of CD9-positive cells among anterior lobe cells was 3.9% ± 0.2% (Fig. 3B). One drop of suspension of the CD9-positive fraction was used for smear preparation and immunocytochemistry. We observed that most of the cells were immunopositive for CD9 (Fig. 3C). *Cd9* mRNA levels were 14.0-fold higher in the CD9-positive cell fraction than in the CD9-negative fraction (Fig. 3D). In addition to the *S100β* mRNA levels, those of *Cd13*, *Cd24*, *Cd81*, *Cd133*, and *Cd184* among the 10 CD antigen genes were also significantly higher in the CD9-positive cell fraction than in the CD9-negative cell fraction (Fig. 3D). Conversely, the mRNA levels of all anterior pituitary hormones (growth hormone [*Gh*], prolactin [*Prl*], thyroid-stimulating hormone-beta [*Tshβ*], luteinising hormone-beta [*Lhβ*], proopiomelanocortin [*Pomc*]) were significantly lower in the CD9-positive cell fraction than in the CD9-negative fraction (Fig. 3D). Major stem/progenitor cell markers (*Sox2*, *Sox9*, *Prop1*, *Cadh1*, *Efnb2*, and *Cxcr4*) in the rat anterior lobe were expressed at significantly higher levels in the CD9-positive cell fraction than in the CD9-negative fraction (Fig. 3D). The mRNA levels of these genes relative to that of *Actb* in the CD9-positive cell fraction are shown in Supplementary Fig. S2.

We cultured isolated CD9-positive cells on a non-coated surface with 10% foetal bovine serum (FBS) at a density of 1.0 × 10^5^ cells/cm^2^ for 72 h, followed by immunostaining for CD9 (Fig. 3E) or *in situ* hybridisation for *Cd9* and double immunostaining for S100β and SOX2 (Fig. 3F). After 72 h of cultivation, the proportion of CD9-positive cells among total cells was 86.3% (Fig. 3E), and that of CD9/S100β/SOX2-positive cells among CD9-positive cells was 88.9% (Fig. 3F).

### Role of CD9 in proliferation

**Figure 4:**
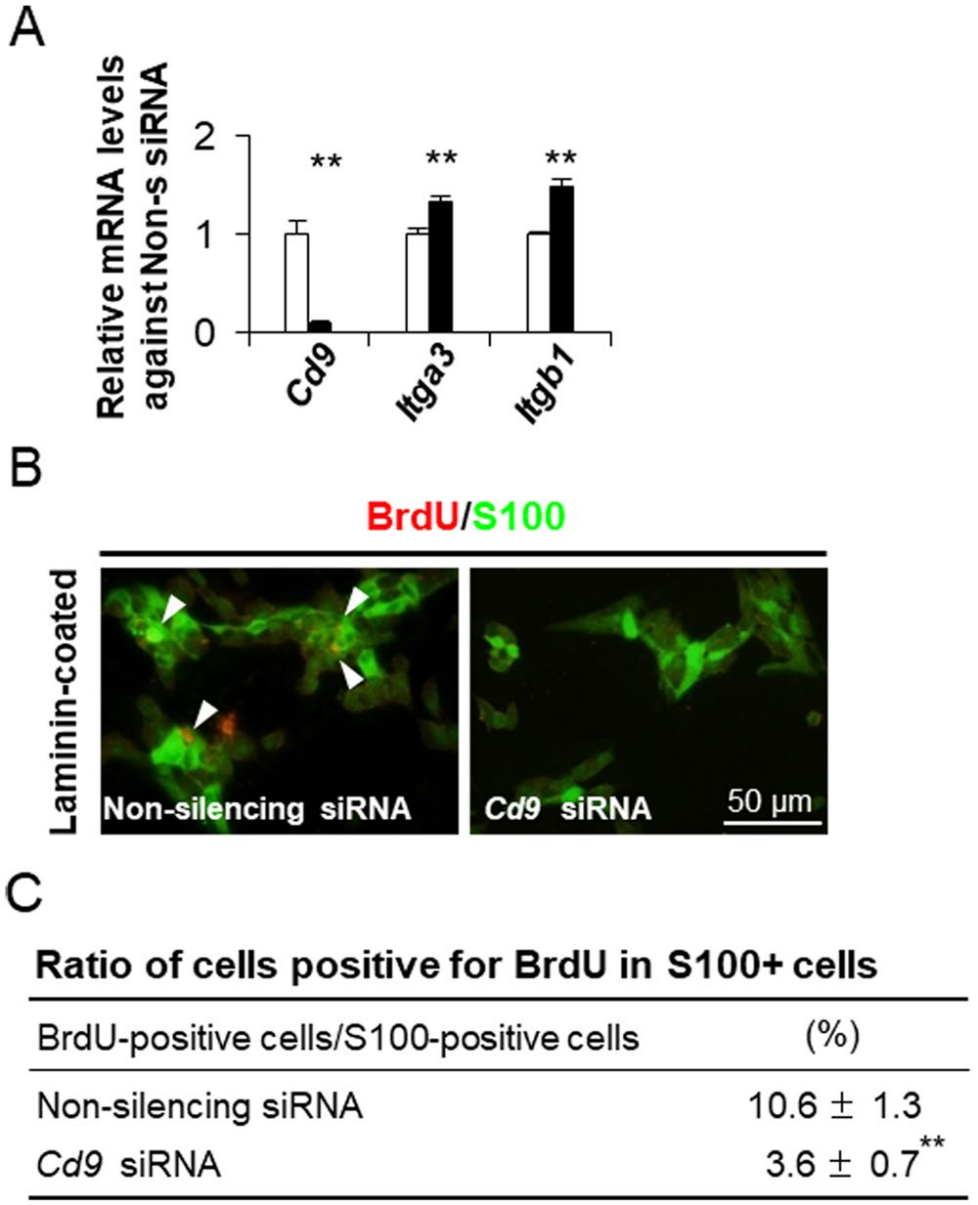
Down-regulation of *Cd9* mRNA levels by siRNA transfection in CD9-positive cells. (**A**) *Cd9*, *Itga3*, and *Itgb1* mRNA levels in CD9-positive cells cultured with non-silencing siRNAs (white bar) or *Cd9-*siRNAs (black bar) for 48 h as determined by qPCR (mean ± SEM, n = 3), followed by normalisation with an internal control (*Actb*). **P < 0.01. (**B**) Merged image of immunocytochemistry of S100β (*green*) and BrdU (*red*) for 24 h after CD9-positive cells were transfected with non-silencing (left) and *Cd9* (right) siRNAs for 48 h and re-plated. (**C**) The ratio of cells immuno-positive for BrdU among CD9-positive cells. **P < 0.01.

CD9 has the ability to associate with various integrins^20,21^. In previous studies, we found that S100β-positive cells exhibited marked proliferation activity under the influence of laminin, an ECM component of the basement membrane, through the ECM receptors integrin-α3 (ITGA3) and β1 (ITGB1)^22,23^. To examine the functional role of CD9, we knocked down *Cd9* gene expression using small interfering RNAs (siRNAs) in CD9-positive cells primary cultured on laminin-coated surfaces at 1.0 × 10^5^ cells/cm^2^. *Cd9* expression levels were successfully down-regulated by siRNA treatment (Fig. 4A). Conversely, the expression levels of *Itga3* and *Itgb1* were up-regulated (Fig. 4A). The number of 5-bromo-2ʹ-deoxyuridine (BrdU)-positive signals in cells treated with *Cd9* siRNA was clearly lower than that in control cells (Fig. 4B). In addition, the percentage of BrdU-positive cells among *Cd9* siRNA-treated cells (3.6% ± 0.7%) was significantly lower (*P* < 0.01) than that among control cells (10.6% ± 1.3%) (Fig. 4C).

### Differentiation of CD9-positive cells into endothelial-like cells

**Figure 5:**
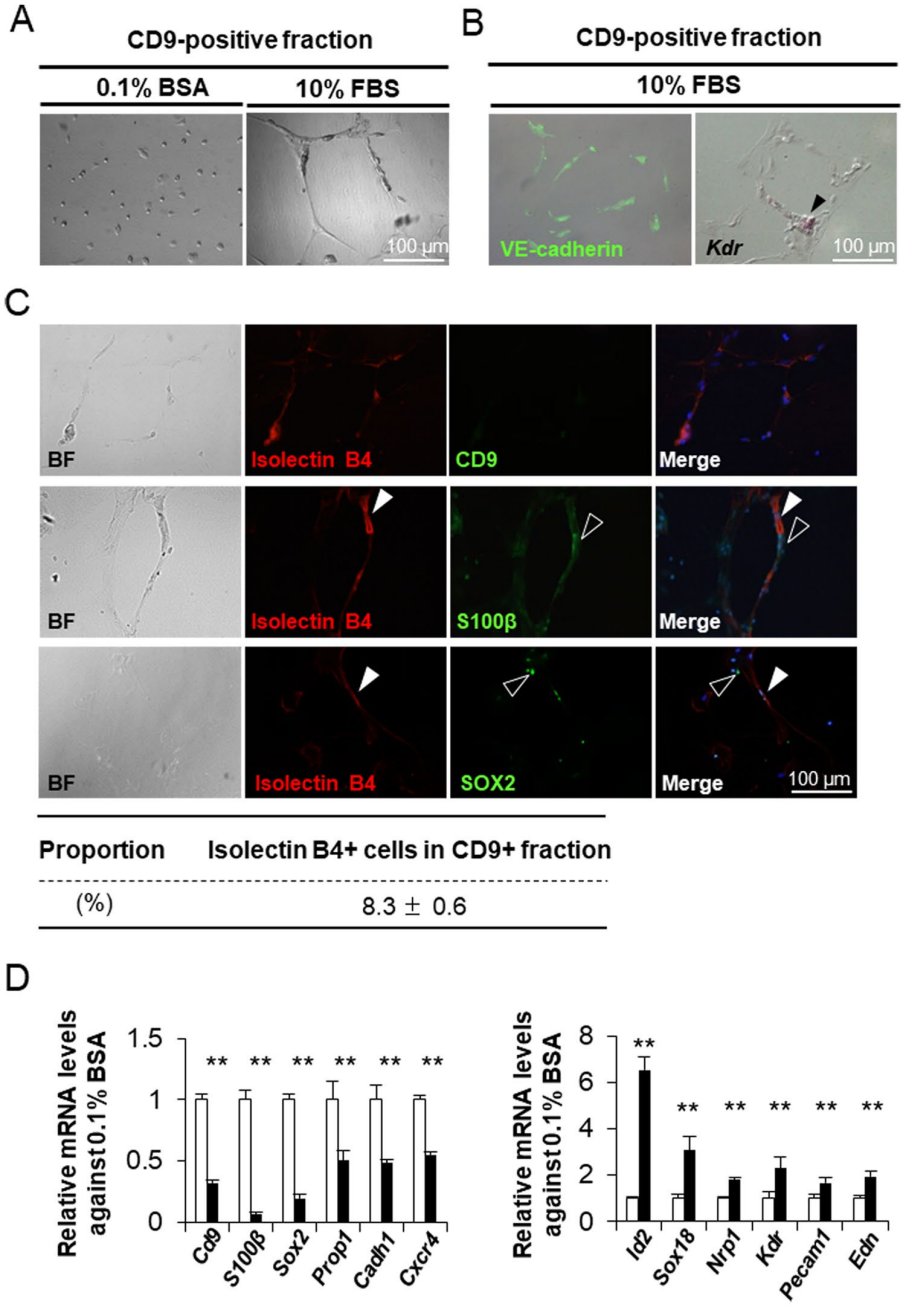
Differentiation of CD9-positive cells into endothelial cells. (**A**) Bright field images of primary cultured CD9-positive cells cultured for 120 h on laminin-coated surfaces in medium with 0.1% BSA (left) or 10% FBS (right) at 2.0 × 10^4^ cells/cm^2^. (**B**) Immunocytochemistry of VE-cadherin (left, *green*) and *in situ* hybridisation of *Kdr* (right, *arrowhead*) after cultivation of CD9-positive cells for 120 h with 10% FBS. (**C**) Double-staining of isolectin B4 with CD9 (upper row), S100β (middle row), and SOX2 (lower row) in rat CD9-positive cells after primary culture for 120 h with 10% FBS. (**D**) Relative ratio of mRNA levels of the following genes after primary culture of CD9-positive cells with 10% FBS (*white bar*) or 0.1% BSA (*black bar*) as determined by qPCR (mean ± SEM, n = 3), followed by normalisation with an internal control (*Actb*): Left graph: *Cd9*, *S100β, Sox2, Prop1, Cadh1*, and *Cxcr4*. Right graph: *Id2, Sox18*, *Nrp1*, *Kdr*, *Pecam1*, and *Edn*.

CD9-positive cells were cultured for 120 h on laminin-coated surfaces with 0.1% bovine serum albumin (BSA) or 10% FBS in Medium 199 at 2.0 × 10^4^ cells/cm^2^. CD9-positive cells cultured with 0.1% BSA were solitary or paired, and some were flattened and showed extension of their cytoplasmic processes a short distance (Fig. 5A). Almost all CD9-positive cells cultured with 0.1% BSA were immunopositive for S100β protein (Supplementary Fig. S3). In contrast, when cultured with 10% FBS, the cytoplasmic processes of CD9-positive cells extended over 100 μm and were interconnected between cells, followed by the formation of a capillary-like network (Fig. 5A). The cells forming the capillary-like network were immunopositive for the endothelial cell marker VE-cadherin and expressed *Kdr*, one of a vascular endothelial growth factor receptor (Fig. 5B). Cells positive for isolectin B4 (an endothelial cell marker) were weakly immunopositive for S100β and SOX2 (Fig. 5C, white arrowhead) but were negative for CD9 (Fig. 5C). This demonstrated that a subset of S100β/SOX2-positive cells differentiated into endothelial cells. However, most S100β-positive or SOX2-positive cells were negative for isolectin B4 (Fig. 5C, white open arrowhead). After primary cultivation of the CD9-positive fraction with 10% FBS for 120 h, an average of 8.3% (ranging from 6.6% to 9.4%) of cultured cells was transformed into isolectin B4-positive cells and formed a capillary-like network. The remaining isolectin B4-negative cells, which were still positive for CD9, S100β, and SOX2, did not form an interconnected architecture. Cultivation with 10% FBS resulted in clear decreases in the expression levels of *S100β* and *Sox2* with moderate reductions in *Cd9*, *Prop1*, *Cadh1*, and *Cxcr4* in comparison with levels in cells cultivated with 0.1% BSA (Fig. 5D). In contrast, a significant increase in expression was observed for several genes, including *Id2* and the endothelial cells markers *Sox18*, *Nrp1*, *Kdr*, *Pecam1*, and *Edn* (Fig. 5D).

**Figure 6:**
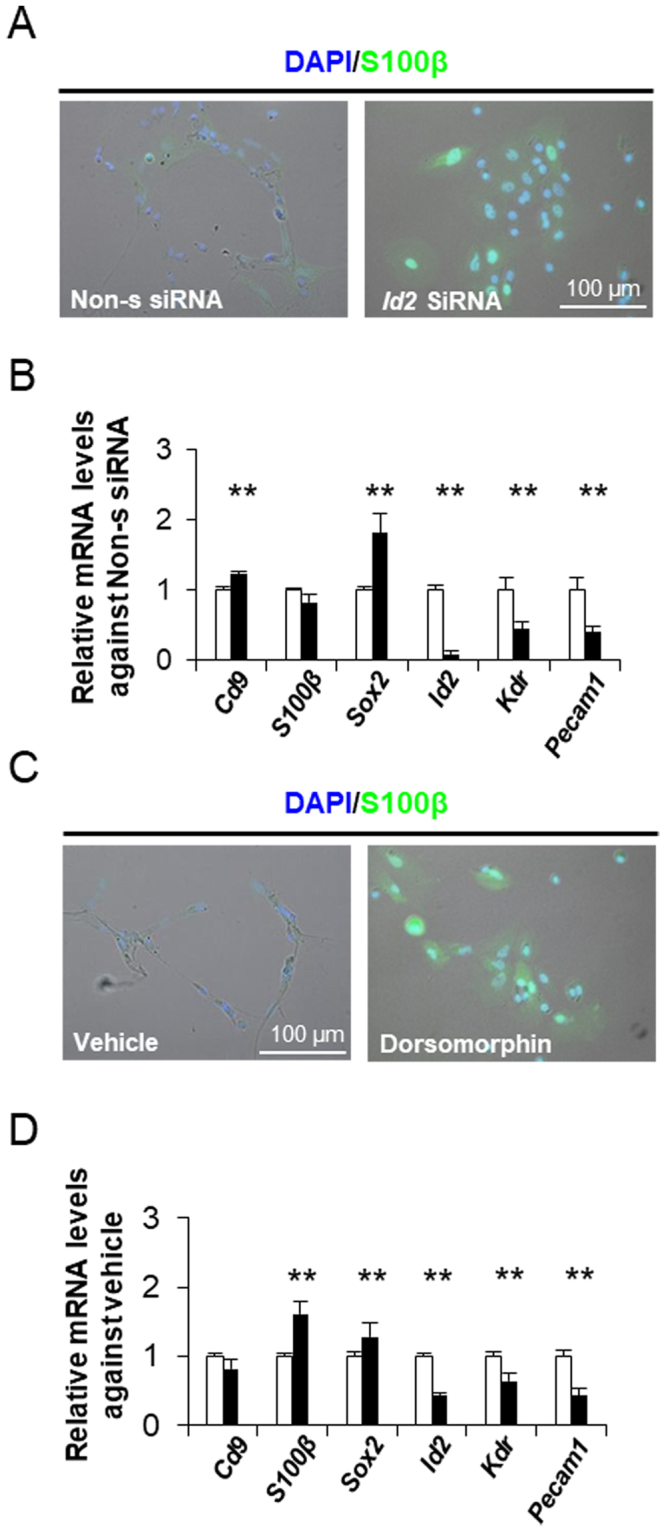
Signalling pathway responsible for endothelial cell differentiation in CD9-positive cells. (**A**) Downregulation of *Id2* by siRNA treatment. Immunocytochemistry of S100β protein (*green*) 96 h following transfection of CD9-positive cells with *Id2* siRNAs. (**B**) mRNA expression levels of *Cd9, S100β, Sox2, Id2, Kdr*, and *Pecam1* 72 h after transfection of CD9-positive cells with *Id2* siRNAs (*black bar*) or non-silencing siRNA (*white bar*; mean ± SEM, n = 3). **P < 0.01. (**C**) Downregulation of BMP signalling by dorsomorphin. Immunocytochemistry of S100β in CD9-positive cells treated for 120 h with vehicle or dorsomorphin. (**D**) mRNA levels after primary culture of CD9-positive cells at 2.0 × 10^4^ cells/cm^2^ for 120 h with vehicle (*white bar*) or dorsomorphin (*black bar*) as determined by qPCR (mean ± SEM, n = 3), followed by normalisation with an internal control (*Actb*). **P < 0.01.

Among these genes, we focused on the expression of inhibitor of differentiation (ID) 2, a transcription factor that contains a helix–loop–helix domain^24^ and is a regulator for differentiation that controls cell fate. Indeed, *in situ* hybridisation and immunohistochemistry showed that CD9-positive cells expressed *Id2* (Supplementary Fig. S4). Notably, since the rapid induction of *Id2* expression by serum stimulation has been reported^24^, the up-regulation of *Id2* expression under the influence of FBS is likely to be a trigger for the differentiation of CD9-positive cells. Hence, we attempted to knock down *Id2* expression using siRNAs in CD9-positive cells cultured on laminin-coated surfaces with 10% FBS. *Id2* siRNA treatment decreased the expression of the target gene and inhibited the formation of the capillary-like network (Fig. 6A). qPCR analysis revealed that *Cd9* and *Sox2* expression levels in *Id2* siRNA-treated cells were higher than those in control CD9-positive cells. However, *Kdr* and *Pecam1* mRNA levels decreased significantly (*P* < 0.01; Fig. 6B). Although *S100β* mRNA levels were not significantly altered, *Id2* siRNA-treated cells were strongly immunoreactive for S100β (Fig. 6A,B). These findings suggested that the up-regulation of *Id2* expression did not directly trigger endothelial cell differentiation in CD9-positive cells.

Next, we analysed the signalling pathway responsible for endothelial cell differentiation in CD9-positive cells. Bone morphogenic protein (BMP) signalling, which exhibits pleiotropic activity in cell differentiation and tissue morphogenesis, is known to be responsible for serum-induced *Id2* expression^24^. CD9-positive cells on laminin-coated surfaces were incubated with vehicle or dorsomorphin, which specifically inhibits the BMP signalling pathway by targeting the type I receptors ALK2, ALK3, and ALK6. As shown in Fig. 6C, dorsomorphin treatment led to a failure in capillary-like formation in CD9-positive cells. Dorsomorphin-treated cells were strongly immunoreactive for S100β (Fig. 6C). Furthermore, the *S100β* and *Sox2* expression levels in the dorsomorphin-treated cells were higher than those in vehicle-treated cells; however, dorsomorphin significantly repressed the expression of *Id2*, *Kdr*, and *Pecam1* (Fig. 6D).

### Vascularisation mediated by CD9/S100β-positive cells in the anterior lobes of prolactinoma model rats

**Figure 7:**
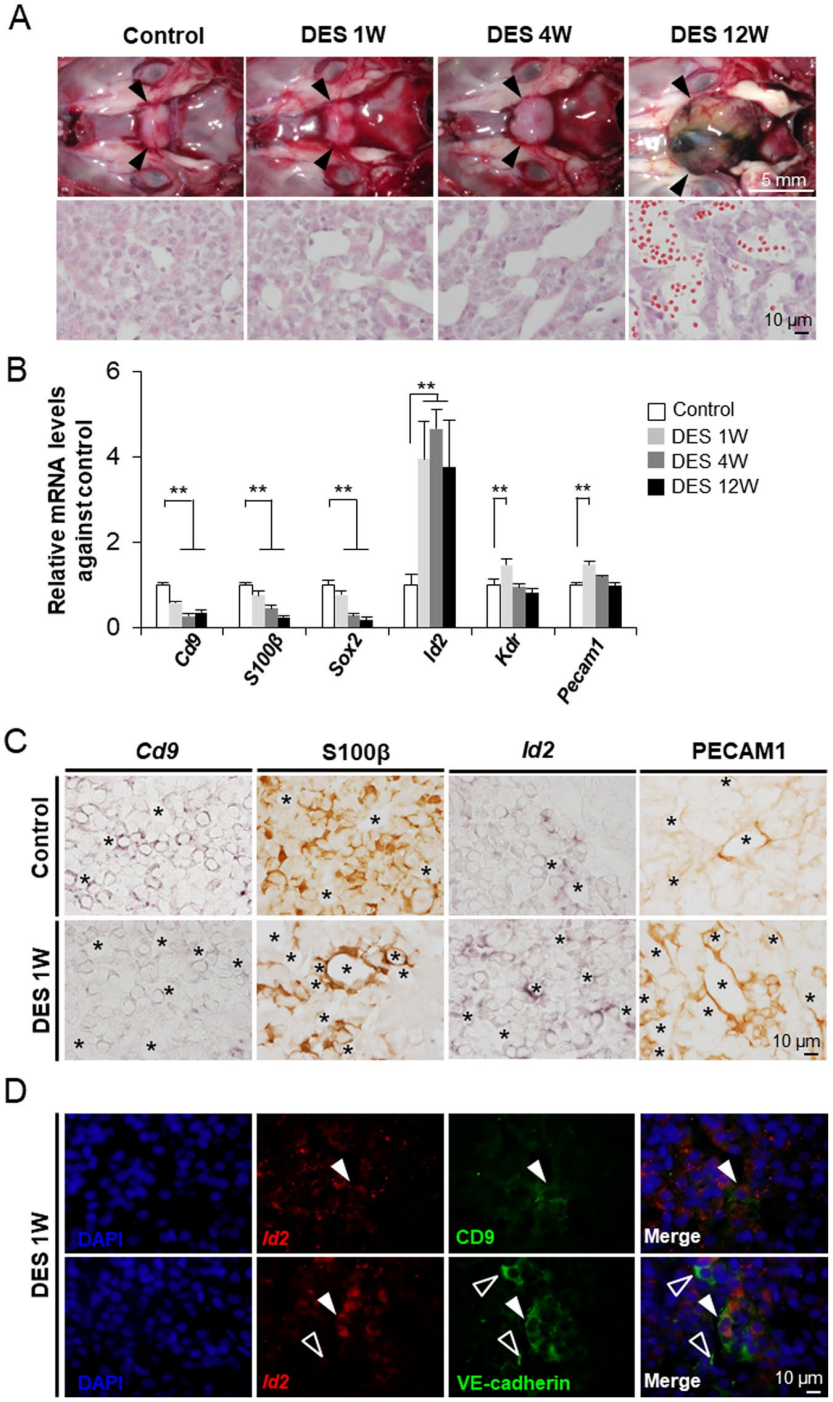
Change in the expression level of *Cd9* in the anterior lobes of prolactinoma model rats. (**A**) The upper row shows images of the pituitary glands of control (Control) rats and those treated with DES for 1 (DES 1 W), 4 (DES 4 W), and 12 weeks (DES 12 W). The lower row shows HE staining of the pituitary glands of control (Control) and male rats treated with DES for 1 (DES 1 W), 4 (DES 4 W), and 12 weeks (DES 12 W). (**B**) *Cd9*, *S100β*, *Sox2, Id2*, *Kdr*, and *Pecam1* mRNA levels after DES treatment as estimated by qPCR (mean ± SEM, n = 3), followed by normalisation with an internal control (*Actb*). **P < 0.01. (**C**) *In situ* hybridisation of *Cd9* and *Id2* and immunohistochemistry of S100β protein and PECAM1 in the anterior lobes of control (Control) and male rats treated with DES for 12 weeks (DES 12 W). Asterisks indicate blood capillaries. (**D**) Double-staining of *Id2* via *in situ* hybridisation and CD9 or VE-cadherin via immunohistochemistry in the anterior lobes of male rats treated with DES for 1 week are shown in the upper and lower rows, respectively. White arrowheads indicate double-positive cells. Open white arrowheads indicate single-positive cells.

To characterise CD9-positive cells under pathological conditions, rats were treated with diethylstilbestrol (DES), establishing a model of prolactinoma, in which an excess amount of prolactin is secreted. Prominent prolactinoma is accompanied by increased pituitary gland weight, high serum prolactin levels^25^, and neovascularisation^26^. After treatment with DES, as shown in Fig. 7A, the tumour mass gradually increased in size in DES-treated rats but not in control rats. In the tumour tissue, neovascularisation was notable, and aggregation of extravascular erythrocytes was often observed among the pituitary cells. We observed many blood vessels in the anterior lobes of DES-treated rats (4–12 weeks of treatment) by HE staining of the tumour tissue: the capillaries were tortuous, and lumens were larger than those in control rats.

The mRNA levels of *Cd9*, *S100β*, and *Sox2* were assessed by qPCR in the anterior lobes of control and DES-treated rats. The results showed a marked decrease in the mRNA levels in DES-treated rats (Fig. 7B). Concomitantly, the levels of *Id2*, *Kdr*, and *Pecam1* mRNAs were significantly increased (*P* < 0.01) in the DES-treated anterior lobes in comparison with those in the control lobes (Fig. 7B). We also observed via *in situ* hybridisation and immunohistochemistry that levels of *CD9* transcripts and S100β proteins were reduced in rats after 1 week of DES treatment (Fig. 7C), followed by further time-dependent decreases (Supplementary Fig. S5). Interestingly, S100β-positive cells in DES-treated rats were localised around blood vessels (Fig. 7C). We examined the localisation pattern of *Id2*-expressing cells and PECAM1-positive cells under the neovascularisation of the anterior lobe in DES-treated rats. These were localised around the blood vessels, and their positive signals were stronger in DES-treated rats (Fig. 7C). In addition, some *Id2*-expressing cells were weakly immunopositive for CD9 and VE-cadherin (Fig. 7D). To determine the direct effect of DES, we cultured CD9-positive cells in the presence or absence of DES with medium containing charcoal/dextran-treated FBS. Regardless of the presence or absence of DES, CD9-positive cells formed capillary-like structures, and *Sox2*, *Id2*, *Kdr*, and *Pecam1* mRNA levels were unchanged (Supplementary Fig. S6). These data indicated that DES has no direct effect on the differentiation of CD9-positive cells to endothelial cells.

## Discussion

The present study succeeded in the isolation of SOX2/S100β-positive stem/progenitor cells from the rat anterior lobe using a monoclonal anti-CD9 antibody and demonstrated that BMP signalling was associated with endothelial cell differentiation in a subset of CD9/S100β/SOX2-positive cells. In addition, the hyperplasia of blood vessels in the anterior lobes of DES-treated prolactinoma model rats was driven in part by the differentiation of CD9/S100β/SOX2-positive cells. Thus, CD9 is a novel indicator that can be used to determine the roles of stem/progenitor cells in the anterior lobe.

S100β-positive cells are known to be composed of heterogeneous subpopulations and to play several biological roles in the pituitary gland. Yoshida *et al*.^12^ showed that the proportion of SOX2-positive cells among S100β-positive cells in the adult rat anterior lobe was approximately 85%. To understand the multiple functions of S100β-positive cells, it is important to be able to isolate S100β/SOX2-positive cells from the anterior lobe in order to utilise several *in vitro* assays. In this study, we showed that SOX2/S100β-positive cells accounted for 84.7% of CD9-positive cells and that the proportion of CD9-positive cells in the anterior lobe was 3.9% according to FACS analysis. These results indicate that the proportion of CD9/SOX2/S100β-positive cells in the anterior lobe was 3.3%. In the CD9-positive fraction, CD9-positive cells accounted for 86%, whereas the proportion of S100β/SOX2-positive cells among CD9-positive cells was about 89%. Therefore, the proportion of CD9/S100β/SOX2-positive cells in the CD9-positive fraction was 77%. These data reflected an enrichment of 23-fold from anterior lobe tissue, indicating that this method provided a powerful tool for investigating the functions of SOX2/S100β-positive cells by utilising CD9 expression.

CD9 was first described as a motility-related factor in 1991, when it was reported that specific anti-CD9 antibody inhibited the migration of multiple cancerous cell lines^27,28^. Thereafter, CD9 has been shown to be associated with various integrins, including α3 and β1, and the migratory functions of CD9 have been attributed to its modulatory activity towards integrin complexes^29^. In previous studies, we found that S100β-positive cells exhibited marked proliferation activity under the influence of ECMs through integrin β1 signalling, which activated the mitogen-activated protein kinase pathway^22^. Downregulation of the *Cd9* gene upregulates the expression of integrins α3 and β1 and inhibits BrdU incorporation in primary culture. These results suggest that the function of CD9 contributes to sustaining proliferation activity through integrin signalling. The downregulation of *Cd9* may trigger the differentiation, rather than the proliferation, of CD9-positive cells under the influence of the ECM.

In the present study, we revealed that approximately 8% of the CD9-positive fraction differentiated into endothelial cells *in vitro* through the involvement of *Id2* expression. These data suggested that S100β/SOX2-positive cells were multipotent progenitor cells and that their differentiation capacity was s influenced by cell culture conditions. Yoshida *et al*. isolated the dense cell clusters originating from the parenchymal niche, termed PS clusters, which are composed of S100β/SOX2-positive cells, from the adult rat anterior lobe. By taking advantage of its tight structure and resistance to protease treatment^11,16^, they showed that S100β/SOX2-positive cells exhibited the capacity for differentiation into endocrine cells in a three-dimensional cultivation system^16^. In contrast, the S100β/SOX2-positive cells in the present study were individually isolated from the whole anterior lobe including the MCL and parenchyma niches by protease reaction and a pluriBead kit with an anti-CD9 antibody. It may be that S100β/SOX2-positive cells outside of PS clusters differentiate into endothelial cells or that the removal of a subset of S100β/SOX2-positive cells from PS clusters using the protease reaction and pluriBead kit triggers their differentiation into endothelial cells. In either case, our findings suggested that S100β/SOX2-positive cells were comprised of heterogeneous subpopulations in the anterior lobe.

To maintain the physiological functions of the pituitary, a blood capillary network composed of endothelial cells and pericytes is essential. We have observed in the developing pituitary gland that S100β/isolectin B4-double positive cells, which may be endothelial-like cells, appear to enter into the embryonic anterior lobe^30^. A portion of S100β-positive cells extend their cytoplasmic processes into the basement membrane around blood capillaries^31^. Some of the CD9/S100β/SOX2-positive cells in the parenchyma may be tissue-resident vascular precursor cells and may participate in vascularisation as suppliers of endothelial cells in the adult anterior lobe. However, we do not currently have definitive data regarding the characteristics of CD9-positive cells. Further studies are necessary to determine whether CD9-positive cells have sphere-forming capacity and differentiate into all types of endocrine cells or whether CD9/S100β/SOX2-positive cells differentiate into endothelial cells *in vivo* using lineage tracing assays.

ID2 functions as a regulator of basic helix–loop–helix transcription factors, and its expression is rapidly induced by serum stimulation of BMP signaling^24^. Lasorella *et al*.^32^ reported that ID2 mediates tumour initiation, proliferation, and angiogenesis in the mouse anterior pituitary. In fact, the present study demonstrated that CD9-positive cells express *Id2* in the rat anterior lobe and that the inhibition of BMP signalling down-regulates *Id2* expression. These observations suggest that the expression of *Id2* contributes to sustaining the plasticity of CD9-positive cells in the niche and that its down-regulation may lead to neovascularisation or tumourigenesis. Indeed, we identified CD9-positive cells in a rat model of prolactinoma, the most common pituitary tumour, accompanied by frequent neo-vasculogenesis in the anterior lobe. DES-treated rats exhibited down-regulation of *Cd9* and stem/progenitor cell markers and up-regulation of *Id2* and endothelial cell markers, as well as the gradual appearance of S100β-positive cells or VE-cadherin-positive/*Id2*-expressing cells around the capillaries of CD9-positive cells. These observations suggest that CD9/S100β/SOX2-positive cells migrate and differentiate into endothelial cells during tumorigenesis. However, the up-regulation of *Id2* expression continued for 12 weeks in the DES-treated anterior lobes, whereas the up-regulation of *Kdr* and *Pecam1* only lasted 1 week. These data led us to speculate that CD9-positive cells may differentiate into endothelial cells via the up-regulation of *Id2* expression in the early stage of DES-treatment, as capillaries were extended and tortuous and haemorrhages were often present in tumour tissues at 12 weeks. Moreover, our results suggested that CD9-positive cell-derived endothelial cells maintained *Id2* expression in tumourigenesis.

In conclusion, we found that *Cd9* was expressed in the majority of S100β/SOX2-positive adult stem/progenitor cells in the rat anterior lobe of the pituitary gland. A subset of CD9/S100β/SOX2-positive cells were shown to differentiate into endothelial cells via stimulation of the BMP/ID2 cascade, indicating that a subset of CD9/S100β/SOX2-positive cells played a role in vascularisation as tissue-resident vascular precursor cells. These CD9-positive cells were observed around the neo-vascular vessels in the pituitary glands of DES-induced prolactinoma model rats. These findings should provide a better understanding of the adult tissue stem cells of the anterior lobe and further preclinical and clinical studies on tumour vascularisation.

## Methods

### Animals

Wistar-crlj S100β/GFP-TG rats that express GFP under the control of the *S100β* promoter were provided by Professor K. Inoue of Saitama University and bred in our laboratory. Male Wistar F344 rats were purchased from Japan SLC, Inc. The day of birth was designated as P0. Eight- to 10-week-old rats weighing 200–250 g were given *ad libitum* access to food and water and housed under a 12-h light/dark cycle. Rats were killed by exsanguination from the right atrium under deep nembutal anaesthesia and were then perfused with Hanks’ balanced salt solution (Thermo Fisher Scientific, Waltham, MA, USA) for culture and FACS or with 4% paraformaldehyde in 0.05 M phosphate buffer (PB; pH 7.4) for HE staining, immunohistochemistry, and *in situ* hybridisation. Oestrogen-induced prolactinoma rats were established from male F344 rats. Eight-week-old F344 rats were subcutaneously implanted with a silastic tube (Kaneka, Osaka, Japan) containing DES (Merck Millipore, Darmstadt, Germany) under ether anaesthesia. Rats were killed at 1, 4 or 12 weeks after implantation with the DES tube. The present study was approved by the Committee on Animal Experiments of the School of Agriculture, Meiji University; Kyorin University; and Jichi Medical University based on the NIH Guidelines for the Care and Use of Laboratory Animals. All experiments were also performed in accordance with the relevant guidelines and regulations of the Committee on Experiments of the School of Agriculture, Meiji University; Kyorin University; and Jichi Medical University.

### Immunohistochemistry and immunocytochemistry

Tissue preparation and immunohistochemistry were performed as described previously^33^. Frozen frontal sections of rat pituitary (8 μm thickness) were obtained using a cryostat (Tissue-tek Polar DM; Sakura Finetek, Tokyo, Japan; CM1860, Leica, Wetzlar, Germany). The standard ABC method was performed using a Vectastain ABC kit (Vector Laboratories, Burlingame, CA, USA) with 3,3ʹ-diaminobenzidine (Dojindo Laboratories, Kumamoto, Japan) as the substrate^33^. Primary and secondary antibodies are listed in Supplementary Table S1. The absence of an observable nonspecific reaction was confirmed using normal mouse, rabbit, or goat serum. Sections were scanned using an epifluorescence microscope (BX61, Olympus, Tokyo, Japan) with the cellSens Dimension system (Olympus).

Cultured cells fixed with 4% paraformaldehyde in 0.025 M PB for 20 min at room temperature (21–23 °C) were first immersed in phosphate-buffered saline (PBS) containing 2% normal goat or donkey serum for 20 min at 30 °C, then incubated overnight with biotinylated isolectin B4 (1:25; Vector Laboratories) or primary antibodies as listed in Supplementary Table S1 at room temperature. After washing with PBS, cells were incubated with secondary antibodies. Biotinylated isolectin B4 was detected by Alexa Fluor 568-conjugated streptavidin (1:400; Thermo Fisher Scientific). The absence of an observable nonspecific reaction was confirmed using normal rabbit or donkey serum. Cells were scanned using a fluorescence microscope (cellSens Dimension system; Olympus).

### cDNA microarray

Dispersed cells from the anterior lobes of S100β/GFP-TG male rats at P5 and P60 were separated into GFP-positive cell fractions by a cell sorter (MoFlo XDP, Beckman Coulter, Fullerton, CA, USA). Total RNA as prepared with TRIzol reagent (Thermo Fisher Scientific) from the GFP-positive cell fraction at P5 and P60 and incubated with RNase-free DNase I (1 U/tube; Promega, Madison, WI, USA). Microarrays were performed on total RNA samples by a custom analysis service (TORAY, Tokyo, Japan) using 3D-Gene (Rat Oligo Chip 20 k). Microarray data were normalised by median levels.

### qPCR

qPCR was performed as described previously^33^. Briefly, qPCR assays were conducted on a Thermal Cycler Dice Real Time System II (Takara, Shiga, Japan) using gene-specific primers and SYBR Premix Ex Taq II (Takara) containing SYBR Green I. Gene-specific primer sequences are listed in Supplementary Tables S2 and S3. For normalisation, levels of β-actin (*Actb*), TATA binding protein (*Tbp*), and glyceraldehyde-3-phosphate dehydrogenase (*Gapdh*) were quantified^34^. The relative gene expression was calculated by comparing cycle times for each target PCR. Cycle threshold values were converted to relative gene expression levels using the 2-^(ΔCt sample −ΔCt control)^ method.

### *In situ* hybridisation

*In situ* hybridisation was performed with digoxigenin labelled cRNA probes, as described in our previous report^33^. DNA fragments were amplified from rat pituitary cDNA using PCR with the primer sets listed in Supplementary Table S2. After *in situ* hybridisation, sections or cells were subsequently stained using immunohistochemistry, as described above. A control experiment using the sense cRNA probe was performed, and no specific signal was detected. Cells were scanned using a microscope (BX61, Olympus).

### Western blotting

Immunoblot analysis was performed as described previously^33^. We applied 20 μg of protein from rat anterior lobes to Ssodium dodecyl sulfate-polyacrylamide gel electrophoresis followed by electrophoretic transfer to an Immobilon-P transfer membrane (Merck Millipore). The membrane was blocked with 5% (w/v) nonfat dried milk for 1 h and incubated overnight with mouse monoclonal antibodies against rat CD9 (0.5 ng/mL; BD Pharmingen, Franklin Lakes, NJ, USA), rat CD24 (0.5 ng/mL; BD Pharmingen) or β-actin (ACTB, 0.1 μg/mL; BioVision, Milpitas, CA, USA) diluted in Can Get Signal Solution (TOYOBO, Osaka, Japan) at room temperature. After washing with Tris-buffered saline with Tween 20 (TBST), the membrane was incubated with horseradish peroxidase (HRP)-labelled secondary antibodies (Envision + System-HRP, anti-rabbit and mouse; DAKO, Glostrup, Denmark) for 1 h. After washing with TBST, specific immunoreactivity was visualised by enhanced chemiluminescence (ECL Plus System, Thermo Fisher Scientific) using an Amersham Imager 600 (Thermo Fisher Scientific).

### FACS analysis

Anterior lobes of male Wistar rats were dissected, and the cells were dispersed as described previously^35^. Rat anterior lobe cells were incubated with anti-mouse CD16/CD32 monoclonal antibody (BioLegend, San Diego, CA, USA) in FACS buffer (Hanks’ balanced salt solution containing 2% FBS) for Fc receptor blocking for 20 min on ice and then incubated with a mouse monoclonal anti-rat CD9 antibody (BD Biosciences) for 30 min on ice. After washing, the cells were incubated with goat anti-mouse IgG antibody conjugated with Alexa Fluor 568 (Thermo Fisher Scientific) for 30 min on ice. The proportion of CD9-positive cells among 7-aminoactinomycin D-negative cells in the anterior lobe cells was analysed on a FACS Aria II Cell Sorter (BD Biosciences) with BD FACSDiva software and FlowJo software (Tree Star, Ashland, OR, USA).

### Isolation of CD9-positive cells

Anterior lobes of male Wistar rats were dissected, and the cells were dispersed as described previously^35^. Dispersed cells were counted using a haemocytometer and separated using a Universal Mouse pluriBeads kit (pluriSelect, San Diego, CA, USA) as described in our previous paper^19^ with a monoclonal anti-rat CD9 antibody (BD Biosciences). CD9-positive and -negative cells were processed for smear preparation, qPCR, cultivation, or immunocytochemistry as described above. After immunocytochemistry, using a 40× objective lens, 10 random fields (157.5 × 210 mm rectangle) were captured per cell culture chamber well containing isolated CD9-positive cells. The populations of CD9-positive cells and CD9/S100β9/SOX2-positive cells were counted using the cellSens Dimension system (Olympus). Three individual experiments were carried out for cell counting.

### siRNA knockdown

CD9-positive cells prepared from Wistar rats were plated at 2.0 × 10^4^ cells/cm^2^ or 1.0 × 10^5^ cells/cm^2^ on 8-well glass chamber slides with laminin-coated surfaces. Cells were then cultured for 24 h in 400 μL Medium 199 with 10% FBS (Merck Millipore) at 37 °C in a humidified atmosphere of 5% CO_2_ and 95% air. For transfection of siRNAs, the culture medium was replaced with 400 μL of Medium 199 with 10% FBS (Thermo Fisher Scientific) supplemented with transfection reagent (INTERFERin at 1:100 v/v; PolyPlus Transfection, Illkirch, France) and siRNAs against *Cd9* mRNA (0.2 μM Rn_*Cd9*_1; Qiagen, Venlo, Netherlands) in wells with cells plated at a density of 1.0 × 10^5^ cells/cm^2^ or siRNAs against *Id2* mRNA (0.2 μM Rn_*Id2*_1; Qiagen) in wells with cells plated at a density of 2.0 × 10^4^ cells/cm^2^, followed by further cultivation for 48 and 120 h, respectively. A non-silencing siRNA without homology to any known mammalian gene was used as a negative control (SI03650325;Qiagen). After cultivation, cells were used for proliferation assays and immunocytochemistry.

### Proliferation assay

To visualise the proliferative activities of cells, the nucleotide analogue BrdU (Merck Millipore) was added at a concentration of 3 μg/mL to the primary culture of CD9-positive cells at a density of 1.0 × 10^5^ cells/cm^2^ for 24 h after adding siRNAs against *Cd9* mRNA. Cells were fixed in 4% paraformaldehyde in 0.025 M PB (pH 7.4) for 15 min at room temperature and were then treated with 4 M HCl in PBS for 10 min. Immunocytochemistry was performed as described above. Ten fields per well were randomly imaged using the cellSens Dimension system (Olympus) installed on a microscope (BX61, Olympus) with a 60× objective lens.

### Differentiation of CD9-positive cells *in vitro*

CD9-positive cells were prepared from Wistar rats as described above and plated on 8-well glass chamber slides with laminin-coated surfaces at a density of 2.0 × 10^4^ cells/cm^2^. Cells were cultured in 400 μL Medium 199 including adenine, adenosine, hypoxanthine, thymine, and additional vitamins, l-glutamine, and phenol red without HEPES (Thermo Fisher Scientific, cat. no. 11150067) with 10% FBS (Merck Millipore) or 0.1% BSA (Sigma-Aldrich, St. Louis, MO, USA) for 120 h at 37 °C in a humidified atmosphere of 5% CO_2_ and 95% air. In the other experimental group, CD9-positive cells among rat anterior lobe cells on laminin-coated slides were treated with medium containing 1.0 × 10^−8^ M dorsomorphin (WAKO, Osaka, Japan), which has been shown to block BMP-mediated signalling, or 10% charcoal/dextran (Merck Millipore)-treated FBS with or without 1.0 × 10^−8^ M DES for 120 h. Dimethyl sulfoxide (DMSO) served as the vehicle. Each observation was performed in triplicate. After cultivation, cells were used for qPCR and immunocytochemistry.

### Statistical analysis

Data are presented as the mean ± SEM of at least three rat preparations for each group. Student’s *t*-tests after F-tests were used for two-group comparisons. The significance of differences between control and test values was determined by Dunnett’s test. A *P* value of less than 0.05 was considered statistically significant.

### Data availability

The datasets generated during and/or analysed during the current study are available from the corresponding author on reasonable request.

## Electronic supplementary material

Supplementary data
