# Supplementary material for: Isolation and characterisation of CD9-positive pituitary adult stem/progenitor cells in rats
Source: Sci Rep. 2018 Apr 3;8:5533. doi: 10.1038/s41598-018-23923-0 (PMC5882946; doi:10.1038/s41598-018-23923-0)

**Supporting Information**

**Isolation and characterization of CD9-positive pituitary adult stem/progenitor cells in rat**

Kotaro Horiguchi^1,2*^, Ken Fujiwara^3^, Saishu Yoshida^2^, Takashi Nakakura^4^, Ken Arae^5^, Takehiro Tsukada^6^, Rumi Hasegawa^1^, Shu Takigami^1^, Shunji Ohsako^1^, Takashi Yashiro^3^, Takako Kato^2^, Yukio Kato^2,7*^

^1^Laboratory for Anatomy and Cell Biology, Department of Health Sciences, Kyorin University, 5-4-1 Shimorenjaku, Mitaka, Tokyo 181-8612 Japan

^2^Institute for Reproduction and Endocrinology, Meiji University, 1-1-1 Higashi-mita, Tama-ku, Kawasaki, Kanagawa 214-8571 Japan

^3^Division of Histology and Cell Biology, Department of Anatomy, Jichi Medical University School of Medicine, 3311-1 Yakushiji, Shimotsuke, Tochigi 329-0498 Japan

^4^Department of Anatomy, Graduate School of Medicine, Teikyo University, 2-11-1 Kaga, Itabashi, Tokyo 173-8605 Japan

^5^Laboratory of Immunology, Department of Health Sciences, Kyorin University, 5-4-1 Shimorenjaku, Mitaka, Tokyo 181-8612 Japan

^6^Department of Biomolecular Science, Faculty of Science, Toho University, 2-2-1 Miyama, Funabashi, Chiba 274-8510 Japan

^7^Department of Life Science, School of Agriculture, Meiji University, 1-1-1 Higashi-mita, Tama-ku, Kawasaki, Kanagawa 214-8571 Japan

*Address all correspondence to:

Y. Kato

E-mail: yukato@meiji.ac.jp

K. Horiguchi

E-mail: kota@ks.kyorin-u.ac.jp

**
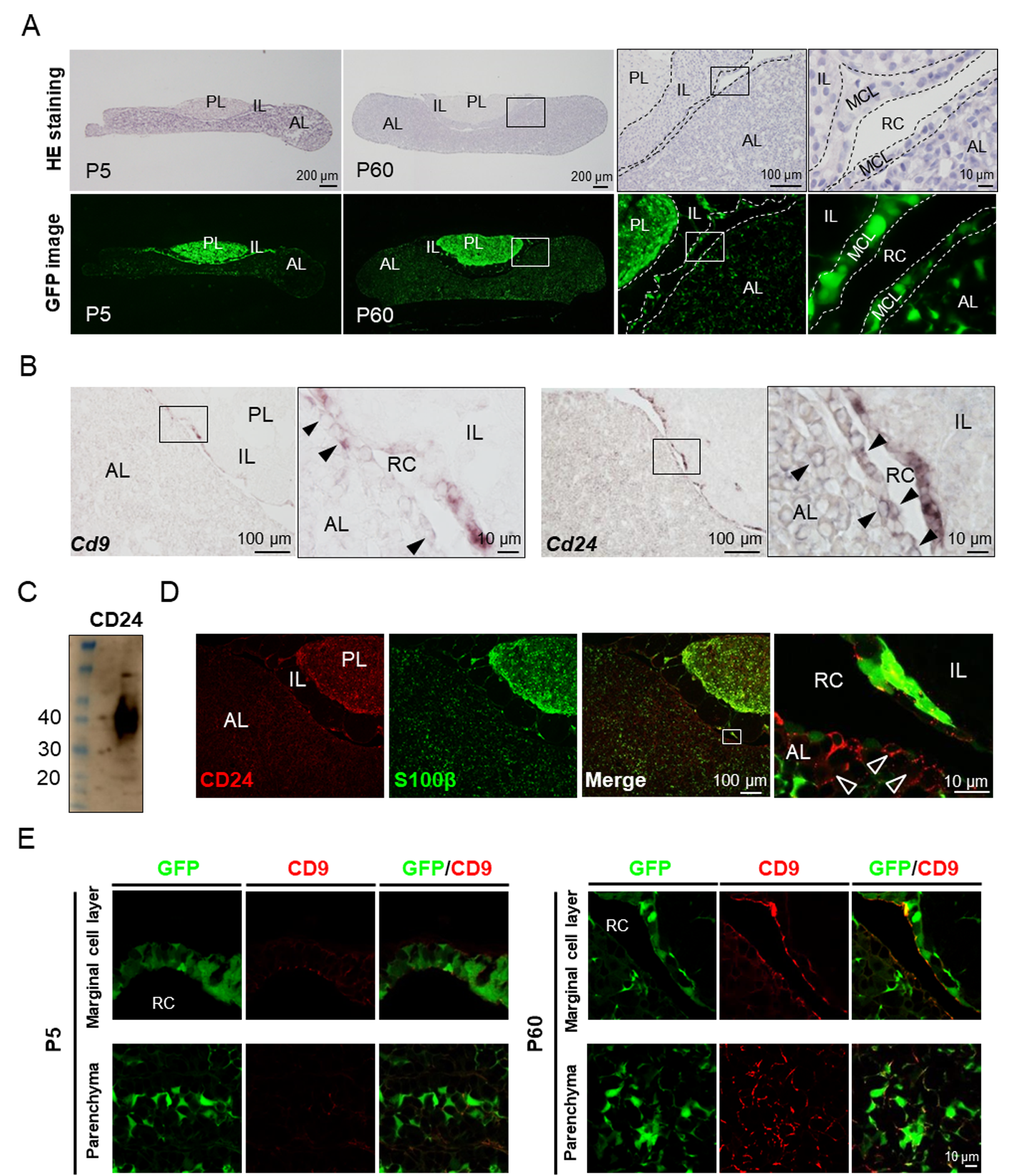
**

**Supplementary Fig. S1.** (**A**) Upper slides: HE staining of pituitary glands of S100β/GFP-TG rats at P5 and P60. Lower slides: GFP image of S100β/GFP-TG rats at P5 and P60. Third images from left in upper and lower rows are high magnifications of boxed areas in left images. Forth images from left in upper and lower rows are high magnifications of boxed areas in third images from left. (**B**) *In situ* hybridisation of *Cd9* (left) and *Cd24* (right). Each right panel is a high magnification of the boxed area in the left panel. Arrowheads indicate positive cells in the anterior lobe. (**C**) Merged images of full-length gels and transfer membranes by western blotting for CD24. Two molecular markers with their molecular weights (kDa) are indicated in the left lane of each panel. The exposure time was 5 min. (**D**) Immunofluorescence staining of CD24 (left) and S100β (second from left). The third panel from the left is a merged image of CD24 and S100β. The forth panel is a high magnification of the boxed area in the third image from the left. Open white arrowheads indicate CD24-positive and S100β-negative cells. AL, anterior lobe; IL, intermediate lobe; PL, posterior lobe; RC, Rathke’s cleft; MCL, marginal cell layer. (E) Immunofluorescence staining of CD9 in marginal cell layer and parenchyma of anterior pituitary from S100β-GFP rats at P5 and P60. RC Rathke’s cleft.


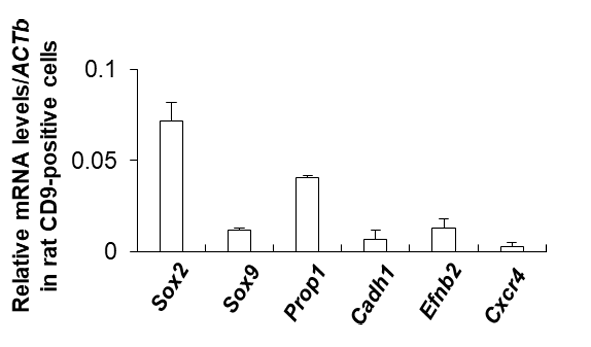


**Supplementary Fig. S2.** mRNA levels of stem/progenitor cell marker genes in CD9-positive cells as determined by qPCR (mean ± SEM, n = 3), followed by normalisation with an internal control (*Actb*).

**
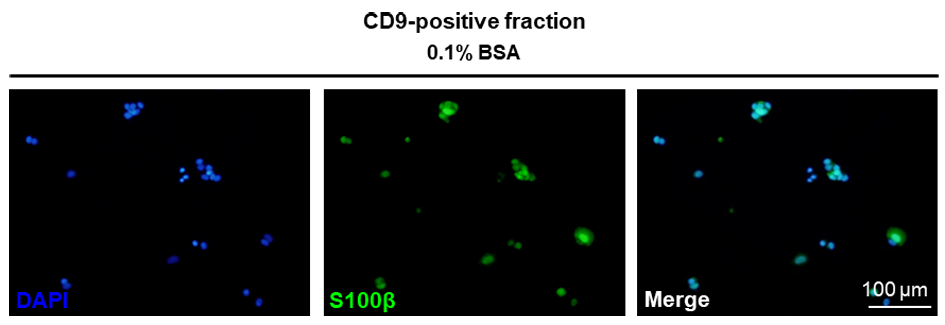
**

**Supplementary Fig. S3.** Immunocytochemistry of S100β after cultivation of CD9-positive cells for 72 h with 0.1% BSA.

**
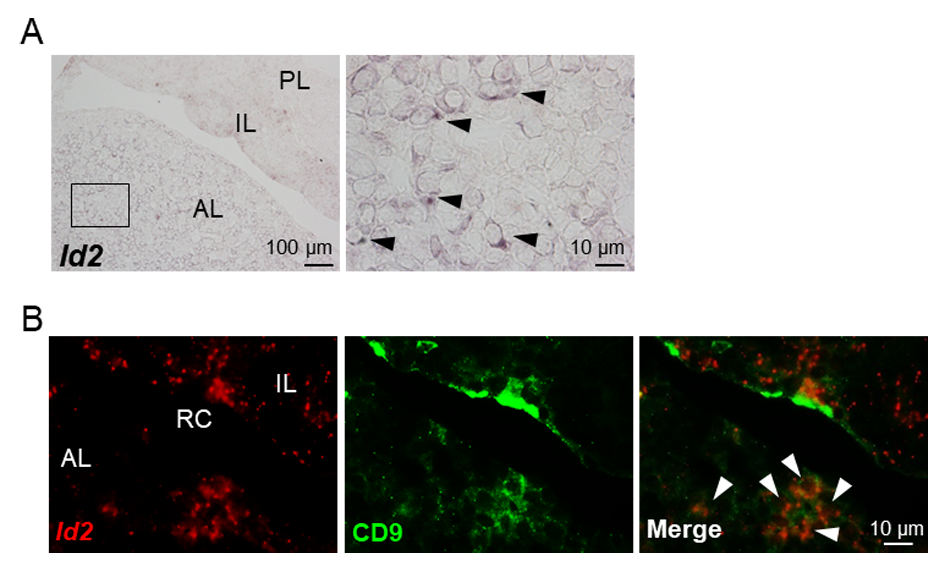
**

**Supplementary Fig. S4.** Expression of *Id2* in the anterior lobes of adult male rats. (**A**) *In situ* hybridisation of *Id2* in the pituitary gland. Left image is a high magnification of the boxed area in the right image. Arrowheads indicate positive signals. (**B**) *In situ* hybridisation of *Id2* and immunohistochemistry for CD9. White arrowheads indicate *Id2*-expressing and CD9-positive cells.

**
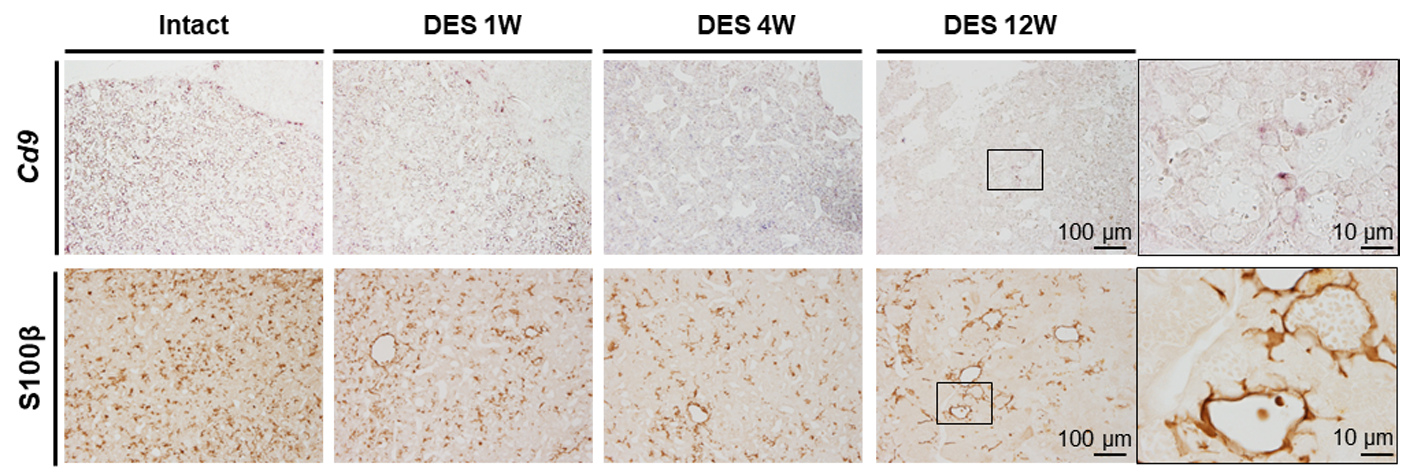
**

**Supplementary Fig. S5.** Upper row: *in situ* hybridisation of *Cd9* in the anterior lobes of control rats (Control) and rats treated with DES for 1 (DES 1W), 4 (DES 4W), and 12 weeks (DES 12W). Lower row: immunohistochemistry of S100β in the anterior lobes of control rats (Control) and rats treated with DES for 1 (DES 1W), 4 (DES 4W), and 12 weeks (DES 12W). Fifth images from the left in the upper and lower rows are high magnifications of the boxed areas in the fourth images from the left.


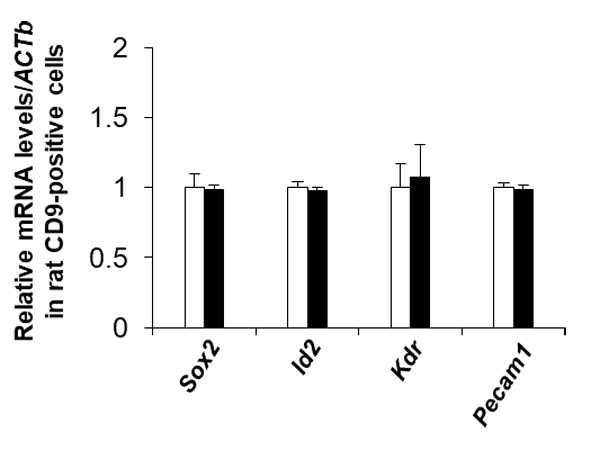


**Supplementary Fig. S6.** *Sox2, Id2, Kdr*, and *Pecam1* mRNA levels in CD9-positive cells 72 h after cultivation in the presence (*white bar*) or absence (*black bar*) of DES with medium containing charcoal/dextran-treated FBS as determined by qPCR (mean ± SEM, n = 3), followed by normalisation with an internal control (*Actb*).

**Supplemental Table S1**


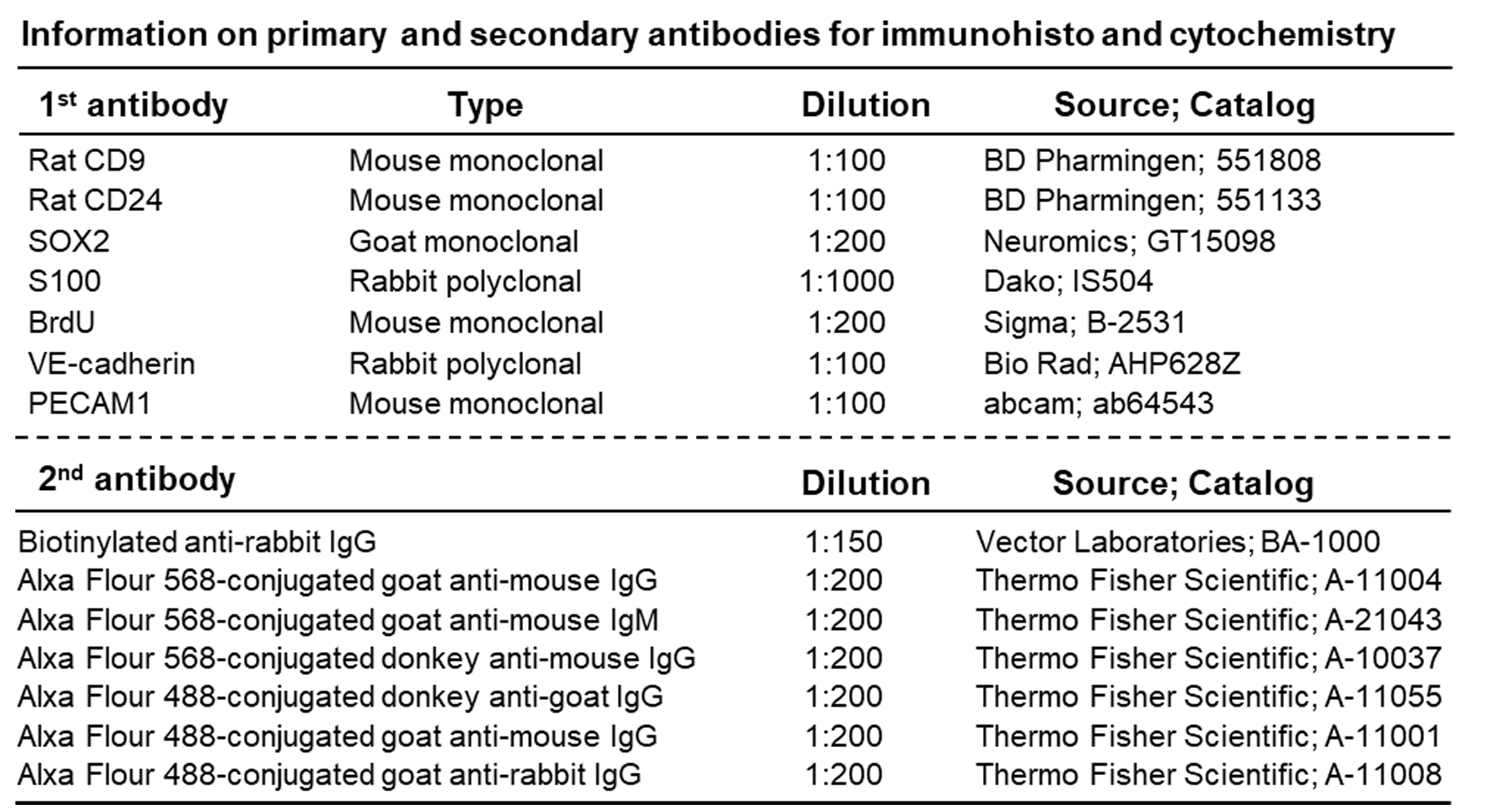


**Supplemental Table S2**


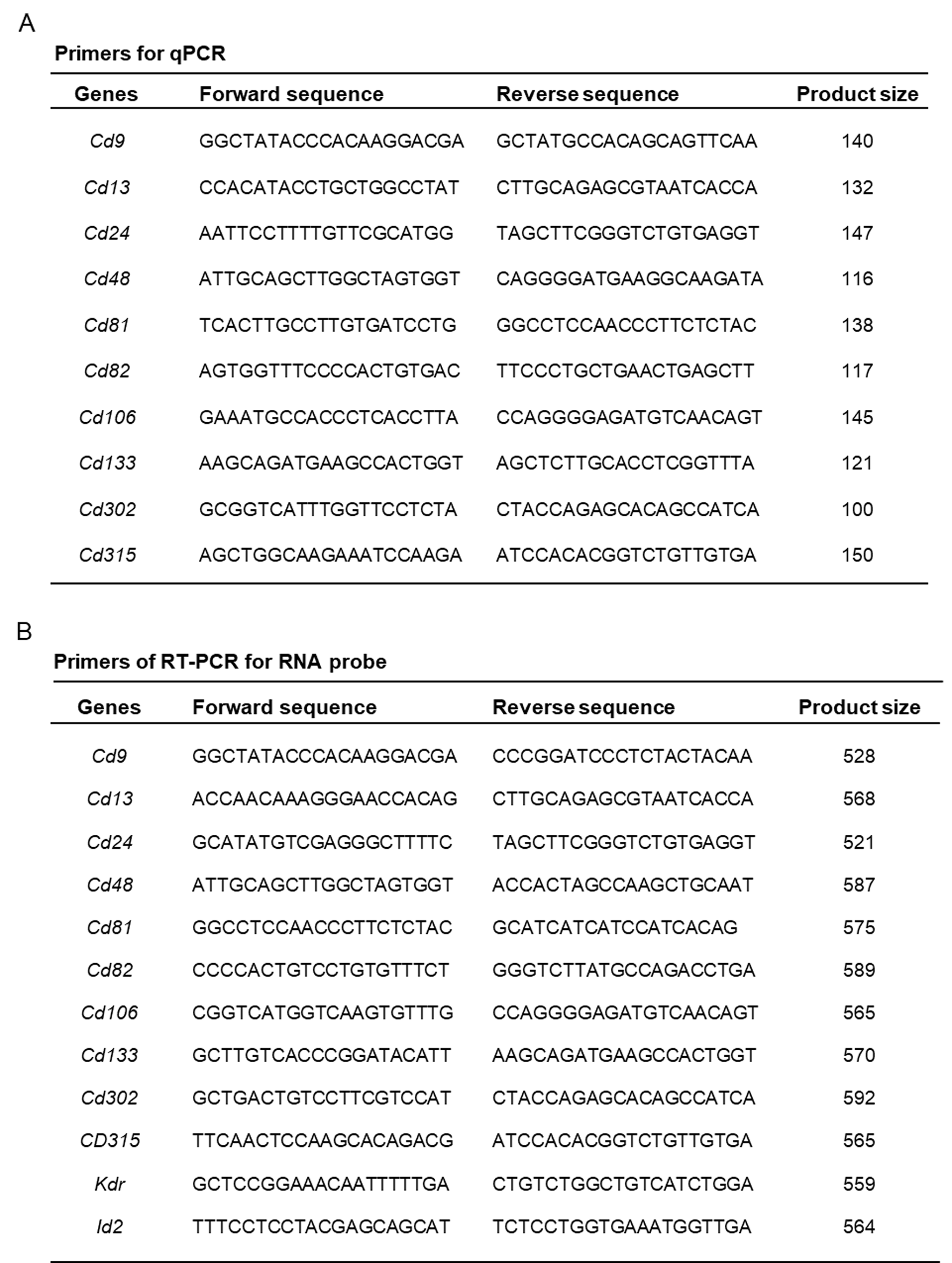


**Supplemental Table S3**


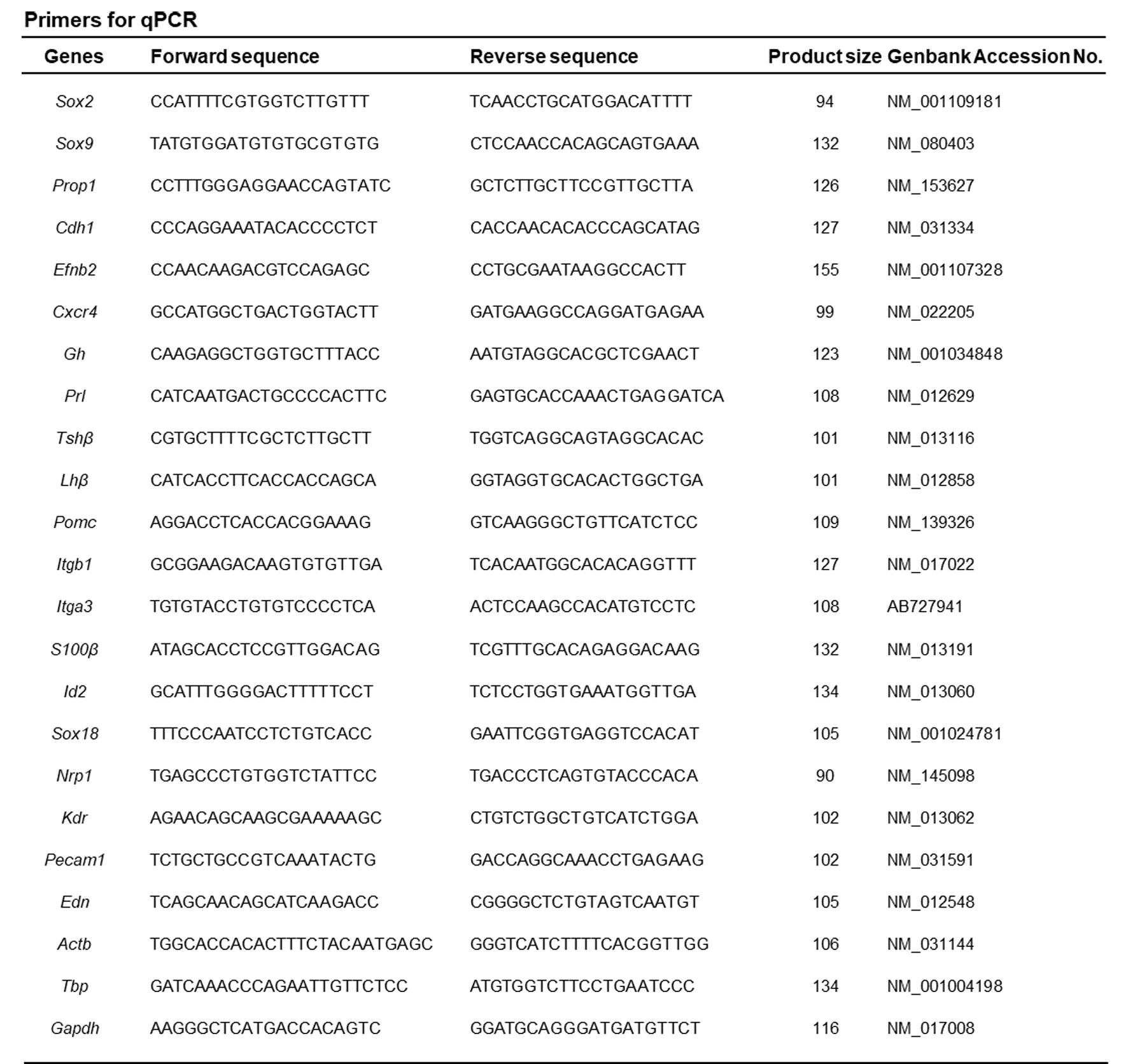

Supplement: Supplementary file 1 — Supplementary data [file 41598_2018_23923_MOESM1_ESM.doc]
